# Lower Prevalence of Antibiotic-Resistant Enterococci on U.S. Conventional Poultry Farms that Transitioned to Organic Practices

**DOI:** 10.1289/ehp.1003350

**Published:** 2011-08-10

**Authors:** Amy R. Sapkota, R. Michael Hulet, Guangyu Zhang, Patrick McDermott, Erinna L. Kinney, Kellogg J. Schwab, Sam W. Joseph

**Affiliations:** 1 Maryland Institute for Applied Environmental Health, University of Maryland, School of Public Health, College Park, Maryland, USA; 2 Department of Poultry Science, Pennsylvania State University, University Park, Pennsylvania, USA; 3 Department of Epidemiology and Biostatistics, University of Maryland, School of Public Health, College Park, Maryland, USA; 4 Center for Veterinary Medicine, Division of Animal and Food Microbiology, Food and Drug Administration, Laurel, Maryland, USA; 5 Department of Environmental Health Sciences, Johns Hopkins Bloomberg School of Public Health, Baltimore, Maryland, USA; 6 Department of Cell Biology and Molecular Genetics, University of Maryland, College Park, Maryland, USA

**Keywords:** antibiotic resistance, antibiotic-resistant bacteria, antimicrobial growth promoter, *Enterococcus*, organic poultry

## Abstract

Background: In U.S. conventional poultry production, antimicrobials are used for therapeutic, prophylactic, and nontherapeutic purposes. Researchers have shown that this can select for antibiotic-resistant commensal and pathogenic bacteria on poultry farms and in poultry-derived products. However, no U.S. studies have investigated on-farm changes in resistance as conventional poultry farms transition to organic practices and cease using antibiotics.

Objective: We investigated the prevalence of antibiotic-resistant *Enterococcus* on U.S. conventional poultry farms that transitioned to organic practices.

Methods: Poultry litter, feed, and water samples were collected from 10 conventional and 10 newly organic poultry houses in 2008 and tested for *Enterococcus*. *Enterococcus* (*n* = 259) was identified using the Vitek_®_ 2 Compact System and tested for susceptibility to 17 antimicrobials using the Sensititre™ microbroth dilution system. Data were analyzed using SAS software (version 9.2), and statistical associations were derived based on generalized linear mixed models.

Results: Litter, feed, and water samples were *Enterococcus* positive. The percentages of resistant *Enterococcus faecalis* and resistant *Enterococcus faecium* were significantly lower (*p* < 0.05) among isolates from newly organic versus conventional poultry houses for two (erythromycin and tylosin) and five (ciprofloxacin, gentamicin, nitrofurantoin, penicillin, and tetracycline) antimicrobials, respectively. Forty-two percent of *E. faecalis* isolates from conventional poultry houses were multidrug resistant (MDR; resistant to three or more antimicrobial classes), compared with 10% of isolates from newly organic poultry houses (*p* = 0.02); 84% of *E. faecium* isolates from conventional poultry houses were MDR, compared with 17% of isolates from newly organic poultry houses (*p* < 0.001).

Conclusions: Our findings suggest that the voluntary removal of antibiotics from large-scale U.S. poultry farms that transition to organic practices is associated with a lower prevalence of antibiotic-resistant and MDR *Enterococcus*.

Antibiotic use in U.S. conventional poultry production poses potential public health concerns with regard to the selection of antibiotic-resistant foodborne bacteria (Institute of Medicine 1998; Levy and Marshall 2004; Silbergeld et al. 2008). In U.S. conventional poultry production, antibiotics are administered for therapeutic, prophylactic, and nontherapeutic purposes (Sapkota et al. 2007; Wegener 2003). Some researchers have estimated that use of antimicrobials in conventional U.S. poultry production (on a per bird basis) increased by 307% from 1985 to the late 1990s, with the use of nontherapeutic antimicrobial growth promoters (AGPs) accounting for a significant portion of this use (Mellon et al. 2001).

The use of AGPs in conventional poultry production selects for resistant bacterial populations in the production environment and retail poultry products (Aarestrup et al. 2000a; Hayes et al. 2001, 2005; Price et al. 2005; Witte 2000). Consequently, the amplification of resistant bacteria in poultry can result in possible increases in the risk of antibiotic-resistant bacterial infections in human populations (Aarestrup et al. 2000a; Hammerum et al. 2010; Levy 1998). Animal-derived antibiotic-resistant bacteria have been shown to spread from animals to humans through direct contact with animals and through the consumption of meat products (Donabedian et al. 2003; van Loo et al. 2007).

These findings are increasingly reported in mainstream news and have become one of the main drivers influencing consumer demand for organic poultry (Oberholtzer et al. 2006), which is perceived to be safer than conventional poultry (Crandall et al. 2009). This consumer demand has spurred increased production of organic poultry, making poultry one of the fastest growing segments of the U.S. organic products sector (Fanatico et al. 2009; Oberholtzer et al. 2006). Retail sales of organic poultry quadrupled between 2003 and 2006 and reached nearly $200 million in 2008 (Oberholtzer et al. 2006).

To accommodate increased consumer demand and to profit from the organic poultry niche, some conventional poultry growers are adopting organic practices and transitioning their conventional farms to certified organic poultry farms (Oberholtzer et al. 2006). These transitions—which include cessation in the use of all antibiotics and agrichemicals (Fanatico et al. 2009)—could result in changes in the prevalence of antibiotic-resistant bacteria on newly organic poultry farms and subsequent organic poultry products. European studies suggest that removing the nontherapeutic use of antibiotics from poultry farms can result in statistically significant reductions in antibiotic-resistant bacteria in animals and food products (Aarestrup et al. 2000b, 2001; Emborg et al. 2003; Hammerum et al. 2007; Heuer et al. 2002; Klare et al. 1999). Reductions in human carriage of resistant bacteria also have been documented in association with antibiotic withdrawals in European poultry production (Klare et al. 1999; van den Bogaard et al. 2000).

However, to date, the studies regarding this issue that have been conducted in the United States have been largely cross-sectional in nature (Han et al. 2009; Price et al. 2007). To the best of our knowledge, no prospective studies have been conducted in the United States to quantify on-farm, temporal changes in antibiotic resistance of foodborne bacteria when antibiotics are removed from U.S. poultry production environments. Voluntary transitions to organic practices among large-scale U.S. poultry producers provide an excellent opportunity to research this issue within the United States. Thus, the objective of this study was to prospectively evaluate the prevalence of antibiotic-resistant enterococci on large-scale conventional poultry farms that transitioned to organic practices. Here we describe the findings from the first year of this study.

## Materials and Methods

*Study sites.* All of the poultry farms participating in this study were located in the Mid-Atlantic United States. Two types of poultry farms were included: large-scale conventional broiler farms that were maintaining conventional practices and using antibiotics (*n* = 5), and large-scale (previously conventional) broiler farms that had just received organic certification and were producing their first flock of certified organic broilers (*n* = 5). All participating farms were operating under the guidance of one feed mill that produced both conventional and certified organic poultry feed. Two individual poultry houses from each farm were included in the study, for a total of 20 poultry houses. Characteristics of the conventional and newly organic poultry houses are summarized in Table 1.

**Table 1:** Characteristics of poultry houses at the time of sampling [mean (range)].

Characteristic	Conventional (*n* = 10)	Organic (*n* = 10)
Months the farm practiced organic methods		0		1.72 (0–3.6)
No. of antibiotics used in feed		3 (2–4)		0
No. of antibiotics used in water		0.18 (0–1)		0
Age of poultry house (years)		15.7 (3–30)		8.8 (3–15)
Length of poultry house (ft)		407 (110–500)		500 (500–500)
Width of poultry house (ft)		44.5 (35–50)		46.8 (44–48)
Months since complete poultry litter change		1.2 (1–1.5)		2.2 (1–3.6)*a*
No. of broiler chicks when flock arrived		30,800 (30,800–30,800)		22,608 (19,300–24,000)
Age of flock (days)		36 (31–40)		36 (29–41)
Cumulative mortality rate (%)		2.51 (1.3–4.3)		4.72 (3–7.5)
Minutes that broilers spent outdoors		0		0*b*
**a**The time since complete poultry litter change is greater than the time the farm practiced organic methods because the farms were not considered “organic” until the first birds to be produced under organic proctices arrived, and these first flocks did not always arrive immediately after the poultry litter cleanout. **b**The organic birds did not go outside although they had the opportunity to do so.				

All of the newly organic poultry houses were certified organic by a state agency accredited by the U.S. National Organic Program (NOP), which promulgates federal organic standards. An overview of common interpretations of the NOP standards that must be met before a poultry farm can be certified organic is provided in Appendix 1.

The specific antimicrobials that were used in feed in the conventional poultry houses were as follows: bacitracin (50 g/ton), virginiamycin (15 g/ton), roxarsone (45.35 g/ton), salinomycin (60 g/ton), nicarbazin (0.0125%), and decoquinate (27.2 g/ton). In addition, gentamicin (GEN) was used at the hatcheries that supplied chicks to conventional poultry houses, and bacitracin, virginiamycin, roxarsone, and salinomycin were used at the breeder facilities that supplied the initial eggs to the hatcheries.

*Sample collection.* From March to June 2008, poultry litter, water, and feed samples were aseptically collected [in sterile Whirl-Pak_®_ collection bags (Nasco, Fort Atkinson, WI)] from the conventional and newly organic poultry houses. Litter samples (500 g) from the top 11–5 cm of litter were collected from three randomly selected areas of each poultry house. Two water samples (500 mL) were retrieved: *a*) one from raw source water before filtration or ultraviolet treatment, and *b*) one from finished water present in the waterlines. One poultry feed sample (300 g) was collected from the central feed hopper within each poultry house. All poultry litter, water, and feed samples were shipped overnight at 4°C and processed within 24 hr.

*Isolation.* Poultry litter and feed samples were enriched in a 1:10 weight-to-volume dilution of 100 mL Enterococcosel Broth (Becton Dickinson & Co., Franklin Lakes, NJ) for 24 hr at 41°C. We included positive and negative control broths for quality control and quality assurance. After 24 hr, 10 μL of the enrichment culture was streaked onto Enterococcosel Agar (EA; Becton Dickinson & Co.) and incubated overnight at 41°C. Presumptive colonies of *Enterococcus* spp.ranged in appearance from brown to black with a brown-black precipitate on EA. Three presumptive *Enterococcus* colonies from each litter and feed sample were streaked onto separate brain heart infusion (BHI) agar plates for purification and incubated at 41°C for 24 hr. A colony was collected from each BHI purification plate and archived at –80°C in Brucella broth with 20% glycerol.

Isolation of *Enterococcus* spp. from water samples was performed in accordance with U.S. Environmental Protection Agency (EPA) Method 1106.1 (U.S. EPA 2006). Briefly, 10-fold dilutions of each water sample were prepared in phosphate-buffered saline (U.S. EPA 2006), and 10 mL of each dilution was filtered through a 0.45-μm, 47-mm mixed cellulose ester filter (Millipore, Billerica, MA). Each filter was placed on a 60-mm plate containing EA, inverted, and incubated at 41°C for 24 hr. Resulting colonies typical of *Enterococcus* spp. were considered presumptive *Enterococcus* spp. Of the recovered presumptive *Enterococcus* spp., three isolates per water sample were purified on BHI and archived in Brucella broth with 20% glycerol at –80°C. Positive (*Enterococcus faecalis* ATCC 29212; ATCC, Manassas, VA) and negative (*Escherichia coli* ATCC 25922) controls were used throughout the isolation process.

*Identification.* All presumptive *Enterococcus* spp. were streaked from archived stocks onto tryptic soy agar amended with 5% sheep’s blood and incubated at 41°C for 24 hr. Presumptive identification of *Enterococcus* spp. was done by Gram staining and testing for catalase production and pyrrolidonyl arylamidase (PYR) activity. All gram-positive, catalase-negative, and PYR-positive isolates were confirmed and identified to the species level using the automated biochemical identification Vitek_®_2 Compact System (BioMérieux Inc., Hazelwood, MO) in accordance with the manufacturer’s specifications.

*Antimicrobial susceptibility testing.* We performed antimicrobial susceptibility testing (AST) on all confirmed *Enterococcus* isolates (*n* = 259) by microbroth dilution using the Sensititre™ system (Trek Diagnostic Systems, Westlake, OH) according to the manufacturer’s directions. Briefly, colonies from pure 18- to 24-hr cultures were transferred to tubes of sterile Sensititre demineralized water (Trek Diagnostic Systems) to achieve a turbidity equivalent to a 0.5 McFarland standard. Then, 50 μL of each suspension was added to sterile Sensititre cation-adjusted Mueller Hinton broth (Trek Diagnostic Systems), and 50 μL of the broth solution was then dispensed into microtiter, gram-positive 96-well plates embedded with 17 test antimicrobials [National Antimicrobial Resistance Monitoring System (NARMS) *Enterococcus* Plate Format; Trek Diagnostic Systems]. Plates were then incubated in the Automated Reading and Incubation System (ARIS; TREK Diagnostic Systems) at 37°C for 18 ± 1 hr. The first 100 plates were read both manually and via the ARIS system for quality assurance comparisons of minimal inhibitory concentration (MIC) determinations. After consistency between the two methods was determined, subsequent samples were read by the ARIS exclusively.

We used Clinical and Laboratory Standards Institute (CLSI) interpretive criteria for microbroth dilution methods (CLSI 2008) to evaluate resulting MICs where breakpoints were available, except for quinupristin/dalfopristin (SYN), for which we used the breakpoint from the European Committee on Antimicrobial Susceptibility Testing (EUCAST 2011). Otherwise, we used the provisional breakpoints used by NARMS (Food and Drug Administration 2009). The following specific antimicrobials (resistance breakpoints) were used: chloramphenicol (CHL; ≥ 32), ciprofloxacin (CIP; ≥ 4), daptomycin (DAP; no interpretive criteria available), erythromycin (ERY; ≥ 8), flavomycin (FLA; ≥ 32), gentamicin (GEN; > 500), kanamycin (KAN; ≥ 1,024), lincomycin (LIN; ≥ 8), linezolid (LZE; ≥ 8), nitrofurantoin (NIT; ≥ 128), penicillin (PEN; ≥ 16), streptomycin (STR; > 1,000), quinupristin/dalfopristin (SYN; ≥ 8), tetracycline (TET; ≥ 16), tigecycline (TIG; no interpretive criteria available), tylosin (TYL; ≥ 32), and vancomycin (VAN; ≥ 32). Multidrug resistance (MDR) was defined as acquired resistance to three or more antimicrobial classes. *Enterococcus faecalis* ATCC 29212, *E. faecalis* ATCC 51299, *Staphylococcus aureus* ATCC 29213, and *Pseudomonas aeruginosa* ATCC 27853 were used as quality control strains.

*Statistical analysis.* We used the generalized linear mixed model (GLMM) method to evaluate associations between the prevalence of antibiotic-resistant *Enterococcus* spp. and poultry production type (conventional or newly organic). The GLMM method was used to account for the clustered nature of the study design, which made it necessary to adjust for intra-poultry house and intra-poultry farm variability. Firth’s bias correction method was used when zero counts occurred for one group (Heinze and Puhr 2010). All statistical analyses were performed using SAS software (version 9.2; SAS Institute Inc., Cary, NC).

## Results

*Prevalence of* Enterococcus *spp. Enterococcus* spp. were isolated from 100% of all conventional and newly organic poultry houses. Poultry litter was the principal environmental media for the recovery of *Enterococcus* spp. from both farm types, with 100% of all litter samples testing positive; however, these microorganisms were also recovered from water and feed samples (Table 2).

**Table 2:** *Enterococcus* spp. isolated from litter, feed, and water samples collected from conventional and newly organic poultry farms.

Total *Enterococcus *isolates [*n* (%)]										
Farm type	*E. durans *	*E. faecalis *	*E. faecium *	*E. gallinarum *	*E. hirae *	Other
Conventional												
Litter (*n* = 90)		1 (< 1)		45 (34)		42 (32)		1 (< 1)		1 (< 1)		0 (0)
Feed (*n* = 29)		0 (0)		10 (7)		15 (11)		1 (< 1)		3 (2)		0 (0)
Source water (*n* = 1)		1 (< 1)		0 (0)		0 (0)		0 (0)		0 (0)		0 (0)
Waterlines (*n* = 13)		0 (0)		0 (0)		12 (9)		0 (0)		1 (< 1)		0 (0)
Total conventional (*n* = 133)		2 (1)		55 (41)		69 (52)		2 (1)		5 (4)		0 (0)
Organic												
Litter (*n* = 95)		6 (5)		63 (50)		18 (14)		4 (3)		3 (2)		1 (< 1)*a*
Feed (*n* = 27)		1 (< 1)		0 (0)		22 (17)		0 (0)		4 (3)		0 (0)
Source water (*n* = 1)		0 (0)		0 (0)		1 (< 1)		0 (0)		0 (0)		0 (0)
Waterlines (*n* = 3)		0 (0)		0 (0)		1 (< 1)		1 (< 1)		0 (0)		1 (< 1)*b*
Total organic (*n* = 126)		7 (6)		63 (50)		42 (33)		5 (4)		7 (6)		2 (2)
**a**Low-discrimination *E. gallinarum*/*faecium*. **b**Low-discrimination *E. durans*/*hirae*.												

Overall, 46% of *Enterococcus* spp. were identified as *E. faecalis* and 43% as *Enterococcus faecium*. *Enterococcus durans*, *Enterococcus gallinarum*, and *Enterococcus hirae* were also isolated from both types of poultry houses in several types of environmental media (Table 2). We found no significant differences in species prevalence between farm types.

*MICs.* MIC ranges, MIC_50_s (MIC for 50% of the bacteria are less than or equal to this MIC) and MIC_90_s (MIC for 90% of the bacteria are less than or equal to this MIC) for *E. faecalis* and *E. faecium* recovered from the poultry houses are shown in Table 3. For *E. faecalis,* 53% of MIC ranges (8 of 15 antibiotics, excluding LIN and SYN because of inherent resistance) differed depending on farm type; for *E. faecium,* 56% of MIC ranges (9 of 16 antibiotics, excluding FLA because of inherent resistance) differed depending on farm type (Table 3). Some MIC ranges differed depending on species (Table 3). Similarly, we also observed some differences in MIC_50_s and MIC_90_s between isolates recovered from different farm types, and between species (Table 3).

**Table 3:** MIC range, MIC_50_, and MIC_90_ (µg/mL) for 17 antibiotics determined for *E. faecalis* and *E. faecium* recovered from conventional (CONV) and newly organic (ORG) poultry farms.

*E. faecalis*					*E. faecium*				
Antimicrobial	Farm type	MIC range	MIC_50_	MIC_90_	MIC range	MIC_50_	MIC_90_
CHL		CONV		4 to ≥ 64		8		8		4 to 16		8		8
		ORG		4 to 16		8		8		≤ 2 to 8		8		8
CIP		CONV		0.5 to 4		1		2		0.25 to ≥ 8		4		4
		ORG		1 to ≥ 8		1		2		0.5 to ≥ 8		2		4
DAP		CONV		≤ 0.5 to 4		1		1		≤ 0.5 to 4		2		4
		ORG		≤ 0.5 to 4		1		2		≤ 0.5 to 4		2		4
ERY		CONV		≤ 0.5 to ≥ 16		≥ 16		≥ 16		≤ 0.5 to ≥ 16		1		8
		ORG		≤ 0.5 to ≥ 16		1		≥ 16		≤ 0.5 to ≥ 16		2		4
FLA*a*		CONV		≤ 1 to ≥ 32		2		8		2 to ≥ 32		≥ 32		≥ 32
		ORG		≤ 1 to ≥ 32		≤ 1		2		2 to ≥ 32		≥ 32		≥ 32
GEN		CONV		≤ 128 to ≥ 2,048		≤ 128		≤ 128		≤ 128 to ≥ 2,048		≤ 128		1,024
		ORG		≤ 128		≤ 128		≤ 128		≤ 128		≤ 128		≤ 128
KAN		CONV		≤ 128 to ≥ 2,048		≤ 128		≥ 2,048		≤ 128 to ≥ 2,048		256		≥ 2,048
		ORG		≤ 128 to ≥ 2,048		≤ 128		≤ 128		≤ 128 to ≥ 2,048		256		256
LIN*b*		CONV		16 to ≥ 64		≥ 64		≥ 64		≤ 1 to ≥ 64		≥ 64		≥ 64
		ORG		16 to ≥ 64		≥ 64		≥ 64		≤ 1 to ≥ 64		16		≥ 64
LZE		CONV		≤ 0.5 to 2		1		2		1 to 4		2		2
		ORG		1 to 4		2		4		1 to 4		2		4
NIT		CONV		8 to ≥ 128		8		64		32 to ≥ 128		≥ 128		≥ 128
		ORG		8 to ≥ 128		16		64		16 to ≥ 128		64		≥ 128
PEN		CONV		2 to ≥ 32		4		8		≤ 0.5 to ≥ 32		16		≥ 32
		ORG		2 to 8		4		8		≤ 0.5 to 16		4		8
STR		CONV		≤ 512 to > 2,048		≤ 512		2,048		≤ 512 to > 2,048		≤ 512		≤ 512
		ORG		≤ 512 to > 2,048		≤ 512		1,024		≤ 512 to > 2,048		≤ 512		≤ 512
SYN*b*		CONV		2 to 32		4		16		≤ 1 to 32		4		8
		ORG		2 to 8		8		8		≤ 1 to 16		2		4
TET		CONV		≤ 4 to ≥ 64		≥ 64		≥ 64		≤ 4 to ≥ 64		≥ 64		≥ 64
		ORG		32 to ≥ 64		≥ 64		≥ 64		≤ 4 to ≥ 64		≤ 4		32
TIG		CONV		0.03 to 0.5		0.12		0.25		0.06 to 0.5		0.12		0.25
		ORG		0.06 to 0.25		0.25		0.25		0.03 to 0.25		0.12		0.25
TYL		CONV		1 to ≥ 64		≥ 64		≥ 64		1 to ≥ 64		4		≥ 64
		ORG		1 to ≥ 64		4		≥ 64		2 to 16		4		8
VAN		CONV		≤ 0.5 to 4		1		2		≤ 0.5 to 2		≤ 0.5		1
		ORG		1 to 4		1		2		≤ 0.5 to 4		1		2
**a***E. faecium* is intrinsically resistant to FLA. **b***E. faecalis* is intrinsically resistant to LIN and streptogramin A (dalfopristin).														

**Figure 1:**
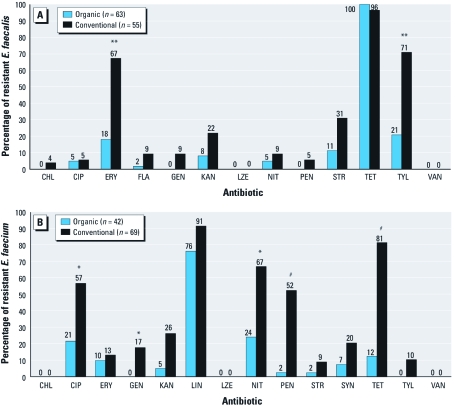
Percentage of *E. faecalis* (*A*) and *E. faecium* (*B*) from conventional and newly organic poultry houses expressing acquired resistance to a particular antibiotic. *E. faecalis* is intrinsically resistant to LIN and streptogramin A (dalfopristin) (Dina et al. 2003); *E. faecium* is intrinsically resistant to FLA. *p < 0.05, **p < 0.01, and ^#^*p* < 0.001, compared with organic poultry houses.

*Acquired antibiotic resistance.* Among *E. faecalis* isolates, acquired antibiotic resistance against nine antimicrobials (CHL, ERY, FLA, GEN, KAN, NIT, PEN, STR, and TYL) was lower among *E. faecalis* from newly organic poultry houses compared with conventional poultry houses (Figure 1A). The differences in percent resistance were statistically significant for ERY (*p* = 0.004) and TYL (*p* = 0.004) (Figure 1A).

Among *E. faecalis*, we observed acquired resistance to CHL, GEN, and PEN only among isolates recovered from conventional poultry houses (Figure 1A). GEN is one of the antibiotics used at the hatcheries that supplied chicks to the conventional poultry houses. We observed no resistance to LZE or VAN among any of the *E. faecalis* recovered from conventional or newly organic poultry houses (Figure 1A). The absence of VAN resistance is most likely attributed to the fact that glycopeptides have never been approved for use in U.S. animal agriculture.

Among *E. faecium* isolates, acquired antibiotic resistance against 11 antimicrobials (CIP, ERY, GEN, KAN, LIN, NIT, PEN, STR, SYN, TET, and TYL) was lower among *E. faecium* from newly organic poultry houses compared with conventional poultry houses (Figure 1B). The differences in percent resistance were statistically significant for CIP (*p* = 0.01), GEN (*p* = 0.047), NIT (*p* = 0.02), PEN (*p* < 0.001), and TET (*p* < 0.001) (Figure 1B).

Among *E. faecium*, we observed acquired resistance to GEN and TYL only among isolates recovered from conventional poultry houses (Figure 1B). We observed no resistance to CHL, LZE, or VAN among any of the *E. faecium* recovered from conventional or organic poultry houses (Figure 1B).

*Sources of antibiotic-resistant bacteria.* Most antibiotic-resistant *E. faecalis* were isolated from poultry litter samples (Table 4). *E. faecalis* isolated from conventional feed samples also expressed acquired resistance to eight antimicrobials (CHL, ERY, FLA, KAN, NIT, STR, TET, and TYL), indicating that conventional poultry feed could be a potential source of exposure to antibiotic-resistant *E. faecalis* among broilers (Table 4). No resistant *E. faecalis* were isolated from organic poultry feed or source water or waterline samples retrieved from either conventional or newly organic poultry houses.

**Table 4:** Antibiotic-resistant *E. faecalis* and *E. faecium* isolated from different environmental sample types recovered from conventional (CONV) and newly organic (ORG) poultry farms.

Resistant *E. faecalis *[*n* (%)]							Resistant *E. faecium *[*n* (%)]						
Antimicrobial	Farm type	Litter	Feed	Source water	Water lines	Litter	Feed	Source water	Water lines
CHL		CONV		0 (0)		2 (20)		0 (0)		0 (0)		0 (0)		0 (0)		0 (0)		0 (0)
		ORG		0 (0)		0 (0)		0 (0)		0 (0)		0 (0)		0 (0)		0 (0)		0 (0)
CIP		CONV		3 (7)		0 (0)		0 (0)		0 (0)		26 (62)		8 (53)		0 (0)		5 (42)
		ORG		3 (5)		0 (0)		0 (0)		0 (0)		9 (41)		0 (0)		0 (0)		0 (0)
ERY		CONV		27 (60)		10 (100)		0 (0)		0 (0)		4 (10)		4 (27)		0 (0)		1 (0)
		ORG		11 (17)		0 (0)		0 (0)		0 (0)		1 (6)		3 (14)		0 (0)		0 (0)
FLA*a*		CONV		4 (9)		1 (10)		0 (0)		0 (0)		21 (50)		14 (93)		0 (0)		8 (67)
		ORG		1 (2)		0 (0)		0 (0)		0 (0)		14 (78)		22 (100)		0 (0)		0 (0)
GEN		CONV		5 (11)		0 (0)		0 (0)		0 (0)		9 (21)		0 (0)		0 (0)		3 (25)
		ORG		0 (0)		0 (0)		0 (0)		0 (0)		0 (0)		0 (0)		0 (0)		0 (0)
KAN		CONV		11 (24)		1 (10)		0 (0)		0 (0)		14 (33)		1 (7)		0 (0)		3 (25)
		ORG		5 (8)		0 (0)		0 (0)		0 (0)		0 (0)		2 (9)		0 (0)		0 (0)
LIN*b*		CONV		45 (100)		10 (100)		0 (0)		0 (0)		40 (95)		15 (100)		0 (0)		8 (67)
		ORG		63 (100)		0 (0)		0 (0)		0 (0)		9 (50)		22 (100)		1 (100)		0 (0)
NIT		CONV		3 (7)		2 (20)		0 (0)		0 (0)		31 (74)		7 (47)		0 (0)		8 (67)
		ORG		3 (5)		0 (0)		0 (0)		0 (0)		7 (39)		3 (14)		0 (0)		0 (0)
PEN		CONV		3 (7)		0 (0)		0 (0)		0 (0)		22 (52)		5 (33)		0 (0)		9 (75)
		ORG		0 (0)		0 (0)		0 (0)		0 (0)		1 (6)		0 (0)		0 (0)		0 (0)
STR		CONV		16 (36)		1 (10)		0 (0)		0 (0)		4 (10)		1 (7)		0 (0)		1 (8)
		ORG		7 (11)		0 (0)		0 (0)		0 (0)		1 (6)		0 (0)		0 (0)		0 (0)
SYN*b*		CONV		7 (16)		10 (100)		0 (0)		0 (0)		8 (19)		5 (33)		0 (0)		1 (8)
		ORG		38 (60)		0 (0)		0 (0)		0 (0)		1 (6)		2 (9)		0 (0)		0 (0)
TET		CONV		43 (96)		10 (100)		0 (0)		0 (0)		36 (86)		11 (73)		0 (0)		9 (75)
		ORG		63 (100)		0 (0)		0 (0)		0 (0)		4 (22)		1 (5)		0 (0)		0 (0)
TYL		CONV		29 (64)		10 (100)		0 (0)		0 (0)		3 (7)		3 (20)		0 (0)		1 (8)
		ORG		13 (21)		0 (0)		0 (0)		0 (0)		0 (0)		0 (0)		0 (0)		0 (0)
**a***E. faecium* is intrinsically resistant to FLA. **b***E. faecalis* is intrinsically resistant to LIN and streptogramin A (dalfopristin).																		

Most antibiotic-resistant *E. faecium* also were isolated from poultry litter samples (Table 4). Antibiotic-resistant *E. faecium* were also recovered from feed and waterline samples from conventional poultry houses and from feed, source water, and waterline samples from newly organic poultry houses (Table 4). Conventional feed was contaminated with *E. faecium* that expressed acquired resistance to 10 antimicrobials (CIP, ERY, KAN, LIN, NIT, PEN, STR, SYN, TET, and TYL), whereas organic feed was contaminated with *E. faecium* that expressed acquired resistance to 6 antimicrobials (ERY, KAN, LIN, NIT, SYN, and TET) (Table 4). No conventional source water samples were contaminated with resistant *E. faecium*, whereas one organic source water sample was contaminated with one LIN-resistant *E. faecium isolate*. Conventional waterline samples were contaminated with *E. faecium* that expressed acquired resistance to 11 antimicrobials (CIP, ERY, GEN, KAN, LIN, NIT, PEN, STR, SYN, TET, and TYL), whereas organic waterline samples were not contaminated with antibiotic-resistant *E. faecium* (Table 4). The differences in waterline contamination between poultry house types could be attributed to the fact that conventional poultry houses, in general, were older than newly organic poultry houses (Table 1), allowing more time for contamination to occur.

**Figure 2:**
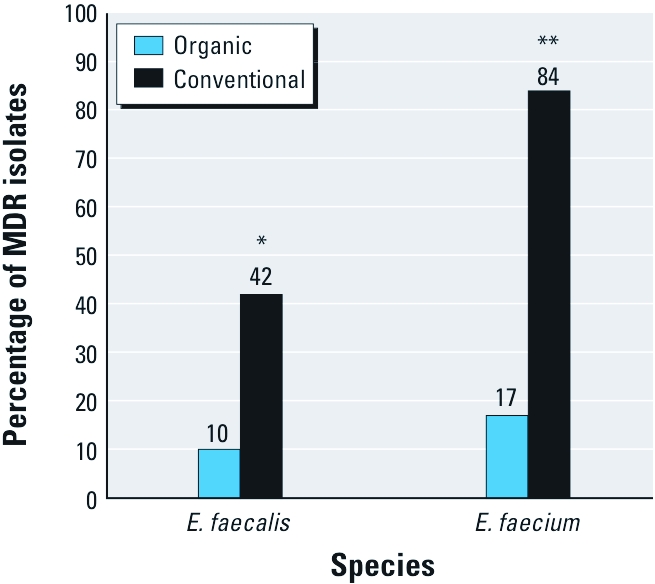
Percentages of MDR *E. faecalis* and *E. faecium* recovered from conventional and newly organic poultry houses *p = 0.02, and **p < 0.001.

*Acquired MDR.* The percentage of MDR *E. faecalis* was statistically significantly lower among isolates from newly organic poultry houses compared with isolates from conventional poultry houses (10% vs. 42%; *p* = 0.02; Figure 2). The percentage of MDR *E. faecium* also was statistically significantly lower among isolates from newly organic poultry houses compared with isolates from conventional poultry houses (17% vs. 84%; *p* < 0.001; Figure 2). Predominant MDR patterns are shown in Table 5.

**Table 5:** Predominant (in > 2 isolates) acquired MDR patterns among *E. faecalis* and *E. faecium* isolated from conventional (CONV) and newly organic (ORG) poultry farms.*a*

Species/farm type		MDR pattern	*n* (%)
*E. faecalis*				
CONV		ERY-GEN-KAN-STR-TET-TYL		3 (5)
		ERY-KAN-STR-TET-TYL		4 (7)
ORG		ERY-KAN-STR-TET-TYL		5 (8)
*E. faecium*				
CONV		CIP-GEN-KAN-LIN-TET-NIT		4 (6)
		CIP-LIN-PEN-TET-NIT		10 (14)
		CIP-LIN-TET-NIT		5 (7)
		LIN-PEN-TET-NIT		3 (4)
		LIN-SYN-TET-NIT		5 (7)
ORG		CIP-LIN-NIT		3 (7)
**a***E. faecalis* is intrinsically resistant to LIN and streptogramin A (dalfopristin) (Dina et al. 2003), and *E. faecium* is intrinsically resistant to FLA; therefore, these species/drug combinations were excluded from the MDR analysis.				

**Figure 3:**
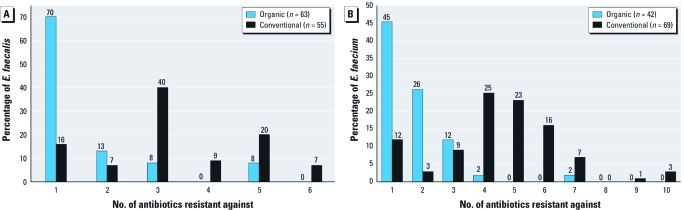
Percentages of *E. faecalis* (*A*) and *E. faecium* (*B*) from conventional and newly organic poultry houses expressing acquired resistance to varying numbers of antibiotics.

The mode number of antibiotics that *E. faecalis* expressed acquired resistance against was one and three, among isolates from newly organic houses and conventional houses, respectively (Figure 3A). The mode number of antibiotics that *E. faecium* expressed acquired resistance against was one and four, among isolates from newly organic houses and conventional houses, respectively (Figure 3B). These findings show that newly organic poultry houses are characterized by individual *E. faecalis* and *E. faecium* isolates that express resistance to fewer numbers of antibiotics compared with their conventional counterparts.

## Discussion

In this study, we observed a significantly lower prevalence of antibiotic-resistant and MDR *E. faecalis* and *E. faecium* on large-scale poultry farms that had just transitioned to organic practices compared with large-scale poultry farms that were maintaining conventional practices. To our knowledge, these are the first U.S. data to show immediate, on-farm changes in antibiotic resistance when antimicrobials are voluntarily withdrawn from large-scale U.S. poultry production.

These findings are in agreement with earlier European and Asian studies that have documented reductions in antibiotic-resistant *Enterococcus* spp. after governmental bans and/or voluntary withdrawals of the use of antibiotics in animal production (Aarestrup et al. 2001; Lauderdale et al. 2007). Using data from the Danish program for surveillance of antimicrobial resistance in bacteria recovered from animals, foods, and humans (Danish Integrated Antimicrobial Resistance Monitoring and Research Programme; DANMAP), Aarestrup et al. (2001) reported significant decreases in the percentages of *E. faecalis* and *E. faecium* resistant to avilamycin, ERY, avoparcin, and virginiamycin, four antibiotics banned by the Danish government for use as AGPs in the late 1990s. For example, from 1997 to 2000 the percentage of ERY-resistant *E. faecium* isolated from broilers decreased from 76.3% to 12.7%, and the percentage of virginiamycin-resistant *E. faecium* isolated from broilers decreased from 66.2% to 33.9% (Aarestrup et al. 2001). In the present study, we observed that the prevalence of *E. faecium* resistant to ERY was 13% and 10% among isolates from conventional and newly organic farms, respectively, whereas the prevalence of *E. faecium* resistant to SYN (a virginiamycin analogue) was 20% and 7% among isolates from conventional and newly organic farms, respectively (Figure 1B).

Reductions in percent resistance to ERY and other antibiotics observed among *Enterococcus* spp. from newly organic poultry farms in the present study may not be as dramatic as those observed by Aarestrup et al. (2001) and other European researchers because poultry houses in the present study were sampled during the production of the first flock of certified organic broilers. Although these poultry houses underwent extensive and comprehensive cleaning events before they could be certified as organic, reservoirs of resistant bacteria may have remained in the packed dirt floor and on fomites within the poultry houses, contributing to persistent low levels of antibiotic-resistant enterococci in newly organic poultry houses. Similarly, Heuer et al. (2002) demonstrated that VAN-resistant enterococci can persist in broiler flocks for > 5 years after antibiotic-selective pressures are removed from the production environment.

Two additional factors likely play significant roles in the persistence of low rates of antibiotic-resistant enterococci observed in newly organic poultry houses in this study. First, U.S. organic certification standards, promulgated through the NOP, apply starting on day 1 of a chick’s life (Fanatico et al. 2009). No organic certification standards need to be met before the first day of life. Thus, some breeder facilities that supply eggs to hatcheries, and hatcheries that ultimately produce “organic” chicks, do not have to meet any organic standards and can therefore use antibiotics among breeder stocks and inject antibiotics into eggs. These practices can result in exposures to antibiotics among “organic” broilers before the first day of life.

Second, organic broilers can be exposed to antibiotic-resistant bacteria through feed and water. Organic poultry feed is required by the NOP to be free of antibiotics, slaughter by-products, and genetically modified organisms (Fanatico et al. 2009). However, our data show that contamination of organic feed with antibiotic-resistant bacteria can occur (Table 4). The question remains as to whether feed is contaminated at the feed mill, during transport, and/or during storage at poultry houses via bioaerosols, insects, rodents, or other factors. Beyond feed, we observed that one source water sample from newly organic poultry houses was contaminated with one LIN-resistant *E. faecium* (Table 4) and one waterline sample from organic poultry houses was contaminated with one MDR *E. gallinarum* (data not shown).

We find it encouraging that the percentages of MDR *E. faecalis* and MDR *E. faecium* in the present study were significantly lower on newly organic poultry farms compared with farms that were maintaining conventional practices. *E. faecalis* recovered from newly organic and conventional farms expressed acquired resistance against a mode number of one and three antibiotics, respectively, and *E. faecium* from newly organic and conventional farms were resistant against a mode number of one and four antibiotics, respectively. These data are in agreement with a recent study by Miranda et al. (2007) that showed that rates of MDR *Enterococcus* spp. were significantly lower among isolates recovered from organic chicken and turkey products compared with conventional products.

As with all field-based studies, the present study had several limitations. As discussed above, we could not control for the fact that organic broilers may have been exposed to antibiotics before the first day of life. This could have influenced the rates of antibiotic resistance observed among *Enterococcus* spp. recovered from newly organic poultry houses; however, because we could not include a control farm that produced chicks that were known to have never been exposed to antibiotics, we could not estimate the contributions of these potential exposures to observed resistance rates. The study is also limited in terms of geographical location. All poultry farms included in this study are located in the Mid-Atlantic United States and under the advisement of one feed mill. Thus, it is unclear whether our results are generalizable across the United States and across the various large-scale contract growers that dominate the U.S. poultry industry. Larger-scale studies based in varying geographical areas at farms managed by different companies are necessary. Finally, this study is limited by the fact that separate conventional poultry farms served as control farms for the newly organic poultry farms. Although it would have been preferable to also sample the newly organic poultry farms before their conversion from conventional to organic practices, this was not possible.

## Conclusions

This study provides the first on-farm U.S. data describing the impacts of eliminating antibiotics from large-scale U.S. poultry production on rates of antibiotic-resistant enterococci. The findings support the hypothesis that removing antibiotic use from large-scale U.S. poultry farms transitioning to organic practices can result in immediate and statistically significant reductions in on-farm antibiotic resistance.

## Correction

In Table 1 of the manuscript originally published online, the value for months since complete poultry litter change was incorrect for organic broilers. It has been corrected here.

## Appendix 1. Overview of Common Interpretations of U.S. NOP Standards for Broiler Production (Fanatico et al. 2009; U.S. Department of Agriculture 2010).

Producer must create and implement an organic system plan and manure management plan.
Broilers must be produced under continuous organic management starting “no later than the second day of life.
All feed components must be organically produced and contain no anti­biotics, other animal drugs, slaughter by-products, or genetically modified organisms.
No antibiotics may be used for animal treatment.
Producer must establish preventative broiler health care practices, and diseases can be prevented with vaccines, bio­security meas­ures, pre­biotics, and pro­biotics.
Maximum stocking densities of broilers is not specified by the NOP, but certifying agencies often require at least 0.14 m2 per bird.
An outdoor access area must be provided to ensure access to fresh air, exercise, and sunlight.
Clean and dry bedding must be provided in an indoor area.
Sanitizers and cleaners used on the property must be on approved products lists.
Agrichemicals cannot be used on the property
